# A Chronic Oral Toxicity Study of Marine Collagen Peptides Preparation from Chum Salmon (*Oncorhynchus keta*) Skin Using Sprague-Dawley Rat

**DOI:** 10.3390/md10010020

**Published:** 2011-12-28

**Authors:** Jiang Liang, Xin-Rong Pei, Zhao-Feng Zhang, Nan Wang, Jun-Bo Wang, Yong Li

**Affiliations:** 1 Department of Nutrition and Food Hygiene, School of Public Health, Peking University, Beijing 100083, China; Email: liangjiangty@163.com (J.L.); rongpx@163.com (X.-R.P.); zhangzhaofeng@126.com (Z.-F.Z.); wn1983117@163.com (N.W.); bmuwjbxy@bjmu.edu.cn (J.-B.W.); 2 Institution for Nutrition and Food Safety, Chinese Center for Disease Control and Prevention, Beijing 100050, China

**Keywords:** marine collagen peptides, chronic oral toxicity, Sprague-Dawley rat

## Abstract

Due to the increased consumption of marine collagen peptides preparation (MCP) as ingredients in functional foods and pharmaceuticals, it was necessary to carry out safety requirements in the form of an oral chronic toxicity assessment. In order to define the oral chronic toxicity of MCP, a 24-month feeding study of MCP was carried out. Sprague-Dawley (S-D) rats at the age of four-week of both sexes were treated with MCP at the diet concentrations of 0%, 2.25%, 4.5%, 9% and 18% (wt/wt). The actual food intake and bodyweight of the individual animals were recorded periodically until sacrifice. Blood and urine samples were collected for serum chemistry evaluations and urinalysis. Throughout the experimental period, there was no toxicologically significant difference between the vehicle and MCP-treated animals with respect to the survival rate, body weight, food consumption, urinalysis, clinical biochemistry parameter and relative organ weight in either sex. Moreover, incidences of non-neoplastic lesions in MCP-treated groups did not significantly increase compared with the control group. Under the present experimental conditions, no higher risk of chronic toxic effects was observed in MCP-treated rats at the diet concentrations of 2.25%, 4.5%, 9% and 18% (wt/wt) than in the rats fed with basal rodent diet.

## 1. Introduction

In recent years it has been recognized that bioactive peptides derived from plant and animal food protein can exert a wide range of physiological or hormone-like biological activities beyond their nutritional value [1]. With marine species comprising nearly one-half of the total global biodiversity, the sea offers enormous resources to be explored for marine bioactive peptides [2,3]. Marine collagen peptides preparation (MCP) is a low-molecular-weight peptide, enzymatically hydrolyzed from the collagen tissues of marine fish. Collagen tissues, including skin, bone and scale account for 30% of marine fish processing waste [4]. As specific protein fragments, MCP has been found to have a wide range of functional and biological properties, including anti-oxidant [4,5], anti-hypertensive [6,7] and anti-skin-aging activities [8]. These bioactive properties or health-enhancing potentials have led to the use of marine collagen peptides as ingredients of functional foods or pharmaceuticals [2,3]. 

Since marine collagen peptides are modified natural products, there are safety requirements to carry out dose-response studies in animals and clinical trials to assess their tolerability and potential chronic adverse effects, which might be exerted by the collagen peptides themselves or their by-products during the processing [1]. Furthermore, for collagen peptides from marine fish, there are significant differences in physicochemical properties and amino acid composition as compared with collagen peptides from livestock such as cow and pig [9,10]. Therefore, previous safety assessment data on collagen peptides from livestock are still insufficient to assess the safety profiles of marine collagen peptides. 

At present time, no data on the oral chronic toxicity of marine collagen peptides *in vivo* has hitherto been available. Considering the increased consumption of marine collagen peptides as ingredients in food and pharmaceuticals, the potential chronic toxicity assessment of this compound is essential [11,12]. Therefore, the 24-month dietary study conducted on Sprague-Dawley (S-D) rats treated with different levels of MCP was carried out to evaluate the chronic oral toxicity profiles of MCP. 

## 2. Results

### 2.1. Survival Rates

**Figure 1:**
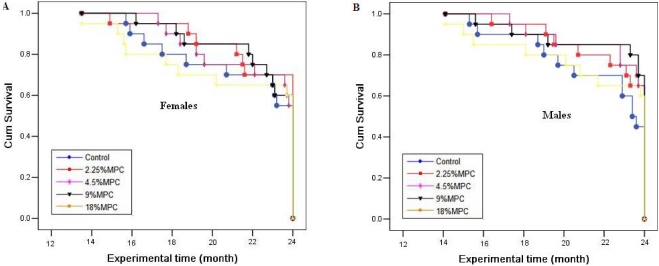
Monthly survival data for Sprague-Dawley (S-D) rats treated with marine collagen peptides preparation (MCP) for 24 months. Values were analyzed for statistical signiﬁcance of differences with Kaplan-Meier survival analysis. All *P* > 0.05 compared with control values.

No mortality was found in any groups treated with marine collagen peptides preparation (MCP) for 12 months. In the 24-month study, the survival rates of 0%, 2.25%, 4.5%, 9% and 18% MCP-treated groups were 55%, 70%, 55%, 60% and 60% for females, and 45%, 65%, 65%, 70% and 60% for males, respectively. There was no statistically significant difference noted in the survival analysis between the control group and the MCP-treated groups (*P* > 0.05) (Figure 1). 

### 2.2. General Condition, Bodyweights, Food Efficiency, Food Consumption and MCP Intake

**Figure 2:**
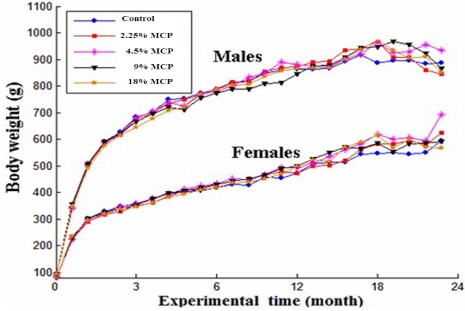
Growth curves for S-D rats treated with marine collagen peptides preparation (MCP) for 24 months. Bodyweight levels were expressed as mean values, and were analyzed by one-way analysis of variance (ANOVA) test. Multiple comparison of Dunnett’s *t*-test was used to evaluate the difference between MCP-treated groups and the control group. All *P* > 0.05 compared with control values.

There was no remarkable difference in general conditions between the vehicle-treated and MCP-treated groups. As shown in Figure 2, there was an age-related tendency to increase bodyweight of the same group. Meanwhile, the bodyweight gain significantly decreased after 18-month MCP treatment. For food consumption, there was a remarkable age-related decreasing trend in each group; while no dose-related change in food consumption was found in either sex when the animals were treated with MCP for 12 months or 24 months. 

Moreover, the food efficiency in the same group significantly decreased with age (Table 1). Throughout the experimental period, since no statistical difference or dose-dependent tendency of body weight and food efficiency was found between the control and MCP-treated groups in either sex, the actual daily intake of the test substance showed a good correlation with its dietary concentrations in different groups. During the 24 months treatment, MCP intake of 2.25%, 4.5%, 9% and 18% MCP-treated groups were estimated to be 1.063, 2.216, 4.609 and 8.586 g/kg·bw/day for females, and 0.907, 1.798, 3.418, 6.658 g/kg·bw/day for males, respectively (Table 2).

**Table 1:** Food efficiency of S-D rats treated with marine collagen peptides preparation (MCP) for 24 months.

Sex	MCP (%)	*n*	6 months Food efficiency (g/100 g)	*n*	12 months Food efficiency (g/100 g)	*n*	18 months Food efficiency (g/100 g)	*n*	24 months Food efficiency (g/100 g)
Female	0	20	3.54 ± 1.41 ^a^	20	2.04 ± 5.80	16	−0.86 ± 5.82	10	−0.02 ± 0.08
	2.25	20	2.21 ± 1.50	20	2.74 ± 6.57	19	0.42 ± 3.15	10	0.04 ± 0.06
	4.5	20	2.74 ± 1.98	20	4.04 ± 2.49	18	−0.35 ± 2.49	10	0.03 ± 0.05
	9	20	3.53 ± 2.24	20	3.50 ± 5.11	19	0.99 ± 2.22	10	0.01 ± 0.04
	18	20	2.74 ± 3.14	20	2.49 ± 1.78	17	0.39 ± 2.32	10	0.04 ± 0.05
Male	0	20	2.13 ± 1.46	20	2.11 ± 3.00	18	0.76 ± 1.46	9	−0.12 ± 0.30
	2.25	20	3.05 ± 2.40	20	4.48 ± 1.66	19	0.57 ± 1.57	10	−0.09 ± 0.14
	4.5	20	2.78 ± 2.86	20	2.79 ± 2.45	19	1.55 ± 2.82	10	0.00 ± 0.02
	9	20	3.33 ± 1.75	20	3.02 ± 2.44	18	−0.50 ± 2.57	10	−0.02 ± 0.07
	18	20	3.97 ± 2.06	20	3.54 ± 3.18	18	0.46 ± 1.53	10	−0.05 ± 0.06

^a^ Values represent the mean ± SD, and the data were analyzed by one-way analysis of variance (ANOVA) test. No significant difference was indicated when compared with the control group.

**Table 2:** Food consumption and intake of marine collagen peptides preparation (MCP) in S-D rat for 12 months and 24 months.

Sex	MCP (%)	*n*	12 months		*n*	24 months	
			Food consumption ^a^ (g/kg·bw/day)	Intake of MCP ^b^ (g/kg·bw/day)		Food consumption ^a^ (g/kg·bw/day)	Intake of MCP ^b^ (g/kg·bw/day)
Female	0	20	65.4 ± 5.7	0.000	10	57.0 ± 3.9	0.000
	2.25	20	63.7 ± 8.9	1.290	10	52.5 ± 4.1	1.063
	4.5	20	65.2 ± 9.2	2.641	10	54.7 ± 7.7	2.216
	9	20	61.4 ± 6.2	4.973	10	56.9 ± 4.9	4.609
	18	20	62.7 ± 7.2	10.157	10	53.0 ± 4.2	8.586
Male	0	20	51.7 ± 4.9	0.000	9	43.7 ± 3.9	0.000
	2.25	20	51.3 ± 4.6	1.039	10	44.8 ± 3.2	0.907
	4.5	20	50.6 ± 5.8	2.049	10	44.4 ± 3.8	1.798
	9	20	50.7 ± 5.8	4.107	10	42.2 ± 3.4	3.418
	18	20	48.8 ± 3.6	7.906	10	41.1 ± 3.5	6.658

^a^ Values represent the mean ± SD. ^b^ Values represent the group means. All *P* > 0.05 compared with control values.

### 2.3. Clinical Biochemical Parameters

As shown in Table 3, no dose-related change or statistical difference was revealed from the serum levels of alanine aminotransferase (ALT), aspartate aminotransferase (AST), albumin (ALB), total protein (TP) and blood urea nitrogen (BUN) after 12-month and 24-month treatment. When animals were treated with MCP for 24 months, the levels of creatinine (CR) non-significantly increased in 9% and 18% male groups as compared with the control group (*P* > 0.05). Additionally, a significant increase in serum TP level was observed in 9% MCP-treated male group (*P* < 0.05), whereas no significant increase of serum TP level was found in female MCP-treated groups *versus* the control group. While the increase of serum TP level in 9% MCP-treated male group was considered to be incidental and not considered treatment-related for the absence of significant dose dependence. 

After 12 months treatment, the significantly decreased serum triglyceride (TG) levels were observed in 2.25% and 4.5% MCP-treated female groups as compared with the female controls (*P* < 0.05), whereas the serum TG levels of male groups did not differ significantly between the control and MCP-treated groups *(P* > 0.05). Moreover, there was no MCP-related effect on the serum levels of total cholesterol (TCHOL) in both sexes (*P* > 0.05). After 24 months of MCP treatment, significantly lower serum levels of TG were observed in 4.5% and 9% MCP-treated female groups as well as in all MCP-treated male groups when compared with the control group (*P* < 0.05). Similarly, the serum levels of TCHOL in 4.5%, 9% and 18% MCP-treated male groups markedly decreased as compared with the control male group (*P* < 0.05), while no significant inter-group difference was observed in the female groups (*P* > 0.05). In addition, no significant difference in the levels of serum glucose (GLU) between the control and MCP-treated groups was revealed from the 12 months and 24 months evaluation data (Table 3). 

### 2.4. Urinalysis

No MCP-related effect was evident in urinalysis parameters and microscopic examination of urine sediment. Urine volume, pH and specific gravity were all within normal limits (data not shown).

### 2.5. Relative Organ Weights

The variability in the organ weights was due to the presence of gross findings such as mass, atrophy, swollen, fibrosis, enlarged and hyperemia area [13]. After 24 months of MCP administration, no significant MCP-related difference was indicated from the relative organ weights of brain, heart, lungs, liver, spleen, kidneys, adrenal glands, testis and ovaries (Table 4). 

### 2.6. Histopathological Examination

Upon histological examination, data of non-neoplastic lesions are presented in Table 5. Most of the lesions related to inflammatory changes and age-related degenerations (fibrosis, emphysema, atrophy and hyperplasia) were found in all groups. Compared with the control group, the incidence of hepatocyte fatty vacuolation decreased markedly in MCP-treated groups of both sexes, while no significance was indicated (*P* > 0.05). No other significantly increased incidence of pathologic change or lesion was observed in MCP-treated groups as compared with the control group (*P* > 0.05).

**Table 3:** Serum biochemical parameters of S-D rats treated with marine collagen peptides preparation (MCP) for 12 months and 24 months.

Months	Parameter		Female MCP (%)					Male MCP (%)				
			0%	2.25%	4.5%	9%	18%	0%	2.25%	4.5%	9%	18%
		*n*	10	10	10	10	10	10	10	10	10	10
12	ALT (U/L)		61.80 ± 12.17 ^a^	59.95 ± 16.43	67.79 ± 18.09	70.27 ± 11.39	68.93 ± 11.59	63.15 ± 16.78	58.40 ± 9.77	65.36 ± 14.71	58.07 ± 19.87	63.21 ± 16.88
	AST (U/L)		126.15 ± 38.96	137.70 ± 31.27	130.38 ± 28.35	134.67 ± 27.07	135.00 ± 43.59	151.10 ± 45.51	134.13 ± 37.31	163.00 ± 42.07	125.50 ± 45.85	123.64 ± 34.70
	ALB (g/L)		37.96 ± 5.24	38.66 ± 2.41	39.49 ± 3.94	39.65 ± 3.12	39.54 ± 3.65	31.64 ± 2.03	31.55 ± 1.48	31.20 ± 2.88	29.37 ± 3.26	31.08 ± 2.01
	TP (g/L)		80.00 ± 4.62	77.70 ± 5.11	80.81 ± 3.99	82.38 ± 5.39	81.20 ± 5.99	70.00 ± 2.81	68.06 ± 6.09	69.50 ± 4.45	65.87 ± 6.33	67.86 ± 2.71
	BUN (mmol/L)		9.36 ± 2.57	8.14 ± 1.71	9.43 ± 1.82	9.93 ± 1.87	9.41 ± 1.38	6.09 ± 1.05	5.56 ± 0.87	6.73 ± 1.68	6.90 ± 2.68	5.86 ± 0.69
	CR (µmol/L)		74.85 ± 13.63	78.65 ± 10.80	75.19 ± 11.86	83.07 ± 12.60	71.60 ± 12.11	68.85 ± 4.90	67.25 ± 3.00	73.86 ± 10.27	75.67 ± 16.39	68.57 ± 3.74
	TCHOL (mmol/L)		2.57 ± 1.63	2.42 ± 0.56	2.43 ± 1.01	2.52 ± 0.39	2.51 ± 1.00	2.95 ± 1.44	2.31 ± 0.33	2.54 ± 1.36	2.67 ± 0.78	2.39 ± 0.67
	TG (mmol/L)		4.14 ± 2.09	2.47 ± 1.64 *	2.87 ± 2.11 *	3.09 ± 1.65	3.22 ± 1.48	2.45 ± 1.35	2.03 ± 1.01	1.81 ± 1.01	2.13 ± 1.54	2.03 ± 0.79
	GLU (mmol/L)		6.27 ± 0.69	6.27 ± 0.52	6.50 ± 0.37	6.66 ± 0.44	6.57 ± 0.40	6.07 ± 1.65	6.25 ± 0.41	6.04 ± 0.62	6.24 ± 0.31	6.39 ± 0.40
24		*n*	10	10	10	10	10	9	10	10	10	10
	ALT (U/L)		41.89 ± 13.93	41.30 ± 5.95	45.30 ± 12.28	43.17 ± 7.14	45.63 ± 15.77	43.50 ± 12.99	41.67 ± 10.97	41.25 ± 7.76	41.89 ± 8.34	43.50 ± 17.13
	AST (U/L)		152.11 ± 26.94	181.70 ± 47.22	162.70 ± 30.79	153.17 ± 40.59	166.13 ± 57.35	164.60 ± 63.55	132.56 ± 43.02	163.25 ± 37.21	160.89 ± 28.94	133.71 ± 50.51
	ALB (g/L)		37.39 ± 2.65	37.38 ± 2.64	35.00 ± 2.12	35.07 ± 1.73	35.94 ± 1.57	28.65 ± 2.47	28.19 ± 2.81	29.20 ± 0.82	29.29 ± 2.82	29.53 ± 1.45
	TP (g/L)		75.11 ± 7.46	78.33 ± 5.10	73.80 ± 6.07	76.50 ± 4.97	74.50 ± 3.51	63.00 ± 4.55	64.11 ± 4.70	61.75 ± 3.86	69.44 ± 5.81 *	67.43 ± 2.57
	BUN (mmol/L)		6.76 ± 1.55	6.41 ± 1.35	6.64 ± 1.59	6.08 ± 2.15	6.21 ± 0.72	6.05 ± 1.46	7.01 ± 4.80	5.08 ± 0.83	5.83 ± 0.87	7.64 ± 4.88
	CR (µmol/L)		67.22 ± 5.14	71.10 ± 5.32	66.90 ± 5.02	72.00 ± 5.34	71.25 ± 5.50	56.90 ± 6.45	58.43 ± 4.12	53.25 ± 2.63	61.10 ± 4.62	61.30 ± 3.01
	TCHOL (mmol/L)		2.61 ± 0.58	2.49 ± 0.42	2.48 ± 0.35	2.79 ± 0.37	2.48 ± 0.45	4.02 ± 1.32	3.49 ± 0.76	2.47 ± 0.27 *	2.98 ± 0.58 *	3.08 ± 0.73 *
	TG (mmol/L)		4.33 ± 1.37	3.63 ± 0.88	2.48 ± 1.11 *	2.96 ± 1.34 *	3.42 ± 1.24	3.99 ± 2.07	2.67 ± 0.85 *	2.21 ± 0.86 *	2.31 ± 1.02 *	2.35 ± 0.87 *
	GLU (mmol/L)		5.27 ± 0.46	5.06 ± 1.09	4.73 ± 0.82	5.58 ± 0.43	4.94 ± 1.15	4.82 ± 0.65	4.63 ± 0.93	5.18 ± 0.57	5.53 ± 0.69	5.37 ± 0.93

^a^ Values represent the mean ± SD. * Significant difference compared with control at *P* < 0.05.

**Table 4:** Relative organ weights of S-D rats treated with marine collagen peptides preparation (MCP) for 24 months.

Sex	MCP (%)	*n*	Brain (g/100 g·bw)	Heart (g/100 g·bw)	Lungs (g/100 g·bw)	Liver (g/100 g·bw)	Spleen (g/100 g·bw)	Kidneys (g/100 g·bw)	Adrenals (g/100 g·bw)	Ovaries (g/100 g·bw)	Testis (g/100 g·bw)
Female	0	11	0.433 ± 0.195 ^a^	0.320 ± 0.070	0.354 ± 0.066	2.808 ± 0.819	0.196 ± 0.232	0.665 ± 0.182	0.034 ± 0.012	0.020 ± 0.010	
	2.25	14	0.450 ± 0.157	0.404 ± 0.096	0.367 ± 0.139	2.638 ± 0.880	0.114 ± 0.059	0.606 ± 0.276	0.030 ± 0.018	0.026 ± 0.010	
	4.5	11	0.519 ± 0.275	0.381 ± 0.118	0.351 ± 0.110	2.387 ± 0.609	0.115 ± 0.046	0.629 ± 0.253	0.025 ± 0.006	0.019 ± 0.008	
	9	12	0.494 ± 0.240	0.430 ± 0.035	0.412 ± 0.094	2.573 ± 0.459	0.111 ± 0.016	0.734 ± 0.290	0.032 ± 0.007	0.026 ± 0.002	
	18	12	0.416 ± 0.099	0.350 ± 0.071	0.348 ± 0.094	2.423 ± 0.849	0.155 ± 0.093	0.659 ± 0.177	0.031 ± 0.007	0.023 ± 0.016	
Male	0	9	0.281 ± 0.082	0.410 ± 0.059	0.391 ± 0.123	3.218 ± 1.216	0.129 ± 0.037	0.726 ± 0.214	0.022 ± 0.009		0.276 ± 0.067
	2.25	13	0.318 ± 0.046	0.433 ± 0.099	0.441 ± 0.079	2.761 ± 0.850	0.134 ± 0.040	0.770 ± 0.246	0.016 ± 0.011		0.384 ± 0.110
	4.5	13	0.369 ± 0.150	0.471 ± 0.079	0.425 ± 0.028	2.468 ± 0.727	0.135 ± 0.087	0.637 ± 0.162	0.017 ± 0.013		0.397 ± 0.135
	9	14	0.338 ± 0.099	0.490 ± 0.110	0.406 ± 0.111	2.870 ± 0.877	0.118 ± 0.072	0.762 ± 0.431	0.019 ± 0.008		0.380 ± 0.090
	18	12	0.382 ± 0.133	0.465 ± 0.088	0.412 ± 0.090	3.199 ± 0.642	0.151 ± 0.052	0.833 ± 0.275	0.015 ± 0.004		0.301 ± 0.098

^a^ Values represent the mean ± SD. All *P* > 0.05 compared with control values.

**Table 5:** Non-neoplastic lesions of S-D rats treated with marine collagen peptides preparation (MCP) for 24 months.

Sites	Lesions	Female MCP (%)					Male MCP (%)				
		0	2.25	4.5	9	18	0	2.25	4.5	9	18
		(*n =* 20)	(*n =* 20)	(*n =* 20)	(*n =* 20)	(*n =* 20)	(*n =* 20)	(*n =* 20)	(*n =* 20)	(*n =* 20)	(*n =* 20)
Brain	Compression	2:00 AM	2	2	1	1	2	1	1	2	2
Pituitary	Hyperplasia, pars distalis	4	3	2	2	3	4	3	2	2	3
Thyroid gland	C-cell hyperplasia	4	3	5	3	2	4	4	3	2	2
Heart	Degeneration myocardial	4	2	2	2	3	4	3	2	2	3
	Focal myocarditis	2	0	1	0	1	0	0	1	0	0
Lung	Inﬂammatory cell foci	4	2	2	1	2	3	2	1	2	2
	Emphysema	5	3	3	3	2	5	3	2	2	3
Liver	Vacuolation, hepatocyte	8	5	3	4	5	7	3	2	2	4
	Fibrosis	1	1	0	2	1	1	0	1	2	0
Stomach	Intestinal metaplasia	1	0	1	0	1	0	1	0	0	1
Intestine	Diverticulum	0	1	0	1	1	1	0	0	0	0
Spleen	Increased extramedullary hematopoiesis	2	0	1	1	0	1	0	1	0	0
Kidney	Fibrosis	2	1	0	1	0	1	2	1	0	1
	Inﬂammatory cell foci, glomerular	7	6	6	5	4	4	3	5	5	3
	Inﬂammatory cell foci, pelvis	1	0	0	1	1	0	1	0	0	1
Adrenal gland	Hyperplasia, cortex	2	2	0	0	1	2	1	1	0	1
	Hyperplasia, medulla	2	0	1	2	1	2	1	0	2	2
Urinary bladder	Transitional cell hyperplasia	1	0	0	1	1	1	2	0	1	0
Mammary gland	Hyperplasia	6	4	5	4	3					
Pancreas	Atrophy, islet cell	6	5	4	4	3	5	4	5	6	4
	Hyperplasia, islet cell	0	1	0	1	1	0	0	1	1	0
Uterus	Metaplasia, squamous	6	5	4	3	4					
	Hyperplasia, endometrium,	10	8	7	9	7					
Ovary	Atrophy	4	2	3	2	1					
Vargina	Cyst, submucosa	1	0	1	1	0					
Testes	Atrophy, testicular						6	5	5	6	4
Prostate gland	Inﬂammation						1	1	0	0	1

^a^ Values represent the number of rats with the lesions. All *P* > 0.05 compared with control values.

## 3. Discussion

With various bioactive properties, marine collagen peptides preparation (MCP) has gained increasing popularity as a dietary nutrition supplement. This long-term feeding study of MCP in S-D rat aims at providing the safety evaluating data. To the best of our knowledge, this is the first chronic toxicity assessment study of the collagen bioactive peptides preparation from Chum Salmon (*O. keta*) skin using S-D rat.

The survival rates in the control group of both sexes were consistent with those reported in previous 24 months feeding studies in S-D rats [14]; this supports the adequateness of our experimental conditions. In the present study, the average bodyweight and food consumption during the 24 months study were not affected by MCP administration, which were in accordance with Wu’s four-week study of collagen peptides from porcine skin [15]. In addition, a previous report had suggested that gelatin-containing combination preparations showed side effects including a sensation of unpleasant taste, occasional pyrosis and eructation [16]. While the data of bodyweight, food consumption and food efficiency in our study indicated that diet added with MCP from Chum Salmon skin might have no significant influence on food appetite and/or absorption in rat. 

No chronic adverse effect or target organ toxicity on liver was revealed from the data of serum biochemistry or histopathological examination of liver. Moreover, there is no previous report available on the hepatotoxicity induced by oral administration of food derived collagen peptides. Regarding the renal toxicity profile of collagen peptides, porcine skin derived collagen peptides at the dose of 16.6 g/kg·bw/day were reported to induce renal hypertrophy in growing rats [15]. However, this study had methodological flaws that instil doubts concerning the conclusion. First, only six male rats/group were allocated for 4-week exposure in this study, which, according to the recent FDA guidelines, was less than optimal. Second, the administration of collagen peptides of 16.6 g/kg·bw/day increased the diet protein content to 34%. As we know, high content of protein may induce renal growth by the induction of insulin-like growth factor-I [17]. So this adverse effect might be due to the high dietary protein content. In our present study, the crude protein in the diet of MCP-treated groups was decreased by 2.25%, 4.5%, 9% and 18%, respectively, to maintain a constant dietary protein level. Thus no clinical adverse effect on kidney function was revealed from the data of serum biochemical parameters including blood urea nitrogen (BUN) and creatinine (CR) and urinalysis in MCP-treated groups throughout the experimental period. In brief, MCP from Chum Salmon skin up to the maximal dose of 18% in the diet in the long-term dietary study does not appear to be toxic to liver and kidney.

Throughout the experiment, there were age-related increasing tendencies in the serum levels of triglyceride (TG) and total cholesterol (TCHOL) in the control group and MCP-treated groups, which were in accordance with other reports [18]. In Wergedahl’s study, the fish protein hydrolysate derived from salmon bone frames was revealed to reduce plasma total cholesterol level and increase the proportion of HDL cholesterol in genetically obese Zucker (fa/fa) rats [19]. It is worth mentioning that incidences of hepatocyte vacuolation in the MCP-treated groups were decreased as compared with the control group. Microscopically, “fatty change” or hepatocyte steatosis is morphologically compatible with hepatocyte cytoplasmic vacuolation, the cell damage caused by lipotoxicity of excessive neutral lipid accumulation in hepatocyte [20,21]. Wergedahl *et al.* found that the rats fed with the hydrolysate of salmon protein had lower lipid content in the liver than those fed with casein, as the former most likely had a higher secretion of triacylglycerol-rich lipoproteins from liver [19]. Moreover, Masataka’s study indicated that peptide from chum salmon or rainbow trout collagen could affect lipid absorption and metabolism in rats and might be useful in suppressing the transient increase of plasma triglycerides [22]. Because of the absence of significant dose dependence, the hyperlipidemic potential of MCP in our study still needs to be verified in further studies. These results of serum TCHOL and TG, however, suggested that long-term administration of MCP is devoid of adverse effect on the lipid absorption and metablism.

## 4. Experimental Section

### 4.1. Preparation and Identification Procedure of Test Substance

Marine collagen peptides preparation (MCP) provided by CF Haishi Biotechnology Ltd. Co. (Beijing, China) was prepared from the skin of wild-caught Chum Salmon (*O. keta*) (from the East China Sea, average body weight, 1.47 kg). The procedure of MCP preparation and identification was according to the method described in our previous research [23]. Briefly, the fish skin was cleaned, scaled, cut into small pieces, defatted and homogenized. After being homogenized and emulsified in distilled water, the material was enzymatically hydrolyzed by complex protease (3000 U/g protein), which including 7% of trypsin, 65% of papain and 28% of alkaline proteinase, at 40 °C and pH 8 for 3 h. The resultant hydrolysate was re-extracted by centrifugation and subsequently separated through ceramic membrane (200 µm). After being purified through a procedure of nanofiltration, condensed by cryoconcentration under vacuum, decolorized with active carbon, filtrated and dried by spray drying, MCP powder used in the following investigations was obtained. 

### 4.2. Characterization of MCP

The MCP sample contains about 90% hydrolyzed protein, 6.0% ash, 1.4% carbohydrate, 2.5% water and 0.1% fat. Then the molecular weight distribution of the samples were analyzed by high-performance liquid chromatography (HPLC, Waters Corp., Milford, MA, USA) and ascertained by LDI-1700 matrix-assisted laser desorption ionization time-of-flight mass spectrometry (MALDI-TOF-MS) (Liner Scientific Inc., Reno, NV, USA).The amino acid composition was analyzed with an amino acid analyzer (H835-50; Hitachi, Tokyo, Japan). In addition, the amino acid composition was further analyzed by an H835-50 automatic amino acid analyzer (Hitachi, Tokyo, Japan). The analysis of MCP indicated 86.5% of the molecular weights distributed between 130 and 1000 Da. The results of amino acid composition in Table 6 show that MCP is rich in Glycine > Glutamic acid > Proline > Hydroxyproline > Aspartic acid > Alanine > Arginine.

**Table 6:** Amino acid composition of marine collagen peptides preparation from Chum Salmon skin.

Amino acid	No. residues/100 residues
Glycine	23.77
Glutamic acid	12.22
Proline	9.79
Hydroxyproline	7.51
Aspartic acid	7.29
Alanine	6.59
Arginine	6.08
Lysine	5.66
Leucine	4.64
Serine	4.23
Valine	2.94
Isoleucine	2.57
Threonine	2.53
Phenylalanine	2.51
Histidine	1.61
Methionine	0.03
Tyrosine	0.03

### 4.3. Experimental Animals and Housing Conditions

Young weaning S-D rats, four-week-old, from both sexes, weighing 80~90 g, were acquired from the Animal Service of Health Science Center, Peking University. Rats were housed two per plastic cages with free access to chow and tap water, and maintained in a filter-protected air-conditioned room with controlled temperature (25 ± 2 °C), relative air humidity (60 ± 5%) and 12 h light/dark cycles (light on 07:30–19:30 h). All animals were handled in accordance with the guidelines of the Principle of Laboratory Animal Care [24] and the guidelines of the Peking University Animal Research Committee.

### 4.4. Experimental Design

After a one-week acclimation period, 200 rats were randomly assigned to five groups (20 animals/sex/group), *i.e.*, vehicle control group and four experimental groups. Control rats were fed with the basal rodent diet (GB14924.3-2001, Vital River Limited Company, Beijing, China). The basal diet contained about 25% protein, 4% fat, 5% fiber and 60% carbohydrate. Rats in the experimental groups were fed with 2.25%, 4.5%, 9% and 18% (wt/wt) MCP in the control diet, respectively. Here, compared with the control diet, 2.25%, 4.5%, 9% and 18% (wt/wt) crude protein in the MCP-added diets of four experimental groups were respectively reduced to keep the constant dietary protein. 

### 4.5. Clinical Investigations

Clinical investigations included observations of daily general condition, bodyweight, food consumption, food efficiency, clinical biochemical examinations and urinalysis.

All animals were observed three times daily, at 08:00, 14:00 and 20:00 h, respectively, for mortality and moribundity. General condition observations included changes in the skin, fur, eyes, somatomotor activity and behavior. 

Animals were single-housed. Specific amount of fresh diet was provided to each animal weekly. The remainder diet and spilled diet were measured weekly to calculate the actual food intakes of individual animal. The food consumption value (g/kg·bw/day) is the ratio of the mean daily food intake of individual animal and mean bodyweight in the individual period of observation. The food efficiency (g/100 g) of individual animals for individual intervals was the average value of bodyweight gain from the per 100 g food intake. The actual food intake and bodyweight of individual animal were recorded weekly in the first six months and every two weeks thereafter until sacrifice. Food consumption and food efficiency calculations for individual intervals and individual animals were summed consecutively to calculate overall food consumption and food efficiency. 

Blood samples for serum chemistry evaluations were collected from the lateral tail vein of the ten survival animals/group/sex every three months during the study. To avoid of selection bias, the first ten animals were selected according to their serial numbers in the order from small to large. The animals were fasted overnight prior to the collection of blood samples. The clinical biochemical parameters included alanine aminotransferase (ALT), aspartate aminotransferase (AST), total protein (TP), albumin (ALB), blood urea nitrogen (BUN), creatinine (CR), triglyceride (TG), total cholesterol (TCHOL) and glucose (GLU), which were determined using an automatic biochemistry analyzer of Olympus AU400 (Olympus Optical Co., Ltd., Tokyo, Japan).

Urine routine examinations were conducted when animals were treated with MCP for 12 months and 24 months. The first ten survival animals of each group were placed in metabolic cages to collect the urine of 24 h. Urine was examined for volume, color, pH and specific gravity, as well as for levels of protein, ketone, urobilirubin, urobilinogen, glycosuria, blood and nitrite. Microscopic examination of the urine sediment was conducted to assess possible changes in the amount of epithelial cells, leukocytes, erythrocytes, cylindrical and crystalline precipitates.

### 4.6. Pathology Examinations

All animals in moribund status during the treatment period were immediately sacrificed by exsanguination and subjected to full necropsies. The skin, all the internal organs, and the cranial, thoracic, abdominal and pelvic cavities were examined to detect any lesion. There were no interim scheduled autopsies. Treated with MCP for 24 months, the remaining surviving animals were all subjected to complete gross necropsy.

At the time of necropsy, the following tissues and organs were collected and fixed in 10% buffered formalin solution: brain, pituitary, heart, lungs, liver, spleen, kidneys, adrenal glands, stomach, intestine, pancreas, urinary bladder, skin, mammary gland, trachea, esophagus, thyroid gland, femoral muscle, epididymis, seminal vesicles, prostate gland, testis, uterus, ovary, vagina and eyes. Then the fixed tissue samples were embedded in paraffin and sectioned at 5 µm. After being stained with hematoxylin and eosin, the slides were examined with an Olympus BH2 microscope (Olympus Optical Co., Ltd., Tokyo, Japan) to detect any lesions. The organs, *i.e.*, brain, heart, lungs, liver, spleen, kidneys, adrenal glands, testis and ovaries of the first ten survival animals/group/sex were trimmed of extraneous fat and weighed to calculate the relative organ weights (the organ to final body weight ratio). Paired organs were weighed together.

### 4.7. Statistical Analysis

Statistical analyses were performed using SPSS (Version 13.0, SPSS Inc.). Variances in the measurement data for bodyweights, food efficiency, food consumption, clinical biochemistry parameters and organ weights were checked for homogeneity by the Bartlett’s test. When the variance data were homogeneous, the one-way analysis of variance (ANOVA) test and multiple comparison of Dunnett’s *t*-test were used. The heterogeneous cases were analyzed with Kruskal-Wallis rank sum test. The results of incidences of pathologic changes or lesions were compared using Chi-square test and the Fisher’s exact probability test. The survival data were analyzed using Kaplan-Meier survival method. The dose-dependent tendency was analyzed using Spearman rank correlation test. A *p* value < 0.05 was considered significant.

## 5. Conclusions

In summary, a chronic toxicity study of MCP from Chum Salmon skin administrated in the diet of S-D rats is reported. No evidence of significant adverse effect or health risk was indicated from the chronic toxicity assessment of MCP up to the diet concentration of 18%, estimated to be 8.586 g/kg·bw/day for females and 6.658 g/kg·bw/day for males. The study results provide an experimental basis for MCP from Chum Salmon skin to be safely used as ingredients of functional foods or pharmaceuticals.
